# Food Insecurity and Diet Quality Among Low‐Income Women in Dhaka City Amidst COVID‐19 and the Ukraine Crisis: A Cross‐Sectional Study

**DOI:** 10.1002/hsr2.72980

**Published:** 2026-08-04

**Authors:** Afsana Rahaman Munmun, Md. Shahadoth Hossain, Sneha Sarwar, Md. Mahbub Alam, Md. Jarif Mahbub, Most Nishat Anam, Zinnia Tasnim, Farah Ahmed, Md. Ruhul Amin

**Affiliations:** ^1^ Department of Food, Bioprocessing and Nutrition Sciences North Carolina State University Raleigh NC USA; ^2^ Department of Nutrition and Food Engineering Daffodil International University Dhaka Bangladesh; ^3^ Institute of Nutrition and Food Science University of Dhaka Dhaka Bangladesh; ^4^ Government College of Applied Human Science Dhaka Bangladesh

**Keywords:** Bangladesh, COVID‐19, diet, food insecurity, nutritional status, slums

## Abstract

**Background and Aim:**

The COVID‐19 pandemic and the Russia–Ukraine war have exacerbated food insecurity and poor diet quality in low‐ and middle‐income countries like Bangladesh. Urban poor populations in Dhaka, particularly slum dwellers, face chronic food insecurity and limited access to nutritious foods. This study assessed food insecurity, diet quality, and nutritional status among women in low‐income slums of Dhaka.

**Methods:**

A community‐based cross‐sectional study was conducted among 400 women residing in low‐income slum communities in Dhaka City of Bangladesh and selected through convenience sampling. Data on socio‐demographics, food insecurity, diet quality, and anthropometry were collected. Food insecurity was assessed using the Food Insecurity Experience Scale (FIES), while dietary diversity and quality were evaluated using the Diet Quality Questionnaire (DQQ). Logistic regression models were used to examine the factors associated with food insecurity and dietary diversity.

**Results:**

The study found a high prevalence of food insecurity, with 90% of participants experiencing moderate or severe food insecurity, of which 34.6% were classified as severely food insecure. Women in the Dhaka South City Corporation had significantly higher odds of food insecurity, while households with multiple earners were less likely to experience it. About 42% of women met the minimum dietary diversity (MDD‐W) criteria. Older age, higher education, high income, and increased food expenditure were positively associated with MDD‐W. Vegetables and pulses were the most frequently consumed foods associated with non‐communicable disease (NCD) prevention. In contrast, sweet foods and deep‐fried items were the most commonly consumed NCD risk foods. Additionally, 8.8% of the women were underweight, and 42.3% were overweight or obese.

**Conclusion:**

This study reveals severe food insecurity, poor dietary quality, and a high prevalence of overweight or obesity among women in low‐income settlements of Dhaka City amid COVID‐19 and the Ukraine Crisis. Targeted interventions, including expanded social safety nets, nutrition education, and regulatory measures to reduce NCD‐risk foods, are essential to improve food access, dietary diversity, and nutritional status in these vulnerable communities during crises.

## 1 Introduction

The COVID‐19 pandemic has severely disrupted economic stability in low‐ and middle‐income countries (LMICs) like Bangladesh, exacerbating challenges related to food security, diet quality, and overall well‐being, particularly among low‐income populations. The low‐income populations have been the most adversely affected group by the negative impacts of the pandemic [1, 2]. As the global economy began to recover, the onset of the Russia–Ukraine war further destabilized food systems by disrupting supply chains, driving up food prices, and limiting access to nutritious food [3, 4]. Recent evidence indicates that the Russia–Ukraine conflict has had widespread implications for developing countries, especially those heavily dependent on imported staple commodities such as wheat, maize, and sunflower oil. For instance, global food prices and availability have been severely affected, exacerbating food insecurity in low‐income nations [5]. Rising food prices have also forced many households in developing contexts to reduce dietary diversity, relying more on calorie‐dense but nutrient‐poor staples at the expense of fruits, vegetables, and protein sources [6].

In Bangladesh, approximately 18.7% of the population lives below the poverty line, with the urban poverty rate at 14.7% [7]. A significant portion of the urban poor reside in slum areas, where 23.3% of residents live in poverty [8]. Even before these global shocks, low‐income communities in Dhaka faced persistent challenges. Inadequate infrastructure, overcrowded living conditions, limited access to clean water, and insufficient healthcare and social safety nets had already contributed to chronic food insecurity and poor diet quality [9, 10]. The Russia–Ukraine war further intensified these challenges by disrupting global supply chains and raising import costs for essential goods, leading to sharp increases in food and fuel prices in Bangladesh [11, 12, 13]. Over the past 5 years, food inflation in Bangladesh has followed a steep upward trend. According to the Bangladesh Bureau of Statistics, food inflation reached approximately 10.72% in January 2025 [14], while the Bangladesh Economic Review 2023–24 reported urban food inflation at around 10.65% and non‐food inflation at 8.87% [15]. This sustained rise in prices has disproportionately burdened urban low‐income households, further constraining their food security and dietary diversity.

Food insecurity, defined as the lack of consistent access to sufficient, safe, and nutritious food necessary for a healthy and active life, remains a major public health concern in Bangladesh. Approximately 9 million individuals are currently experiencing acute food insecurity, primarily due to rising food prices and reduced income opportunities [16]. The “State of Food Security and Nutrition in the World 2023” report highlights that over 52.7 million Bangladeshis face some degree of food insecurity [17]. Evidence from urban slums further highlights the disproportionate burden on low‐income households, with a recent study reporting that 38% of adults in these settings experience severe food insecurity [18]. Food security is intrinsically linked to diet quality [19], and the affordability of a healthy diet is largely dependent on income levels [17]. Data from the Household Income and Expenditure Survey (HIES) 2016 revealed that staple foods constituted 70.4% of the average daily energy intake in Bangladesh, underscoring the poor dietary diversity of the population [20]. A recent study found that nearly 43% of households cannot afford a nutritionally adequate diet, often prioritizing starchy staples over nutrient‐rich foods [21]. The situation is even more severe in slum areas, where dietary diversity remains low due to financial constraints and limited access to diverse food groups [22, 23].

The dual shocks of the COVID‐19 pandemic and the Ukraine crisis have significantly altered dietary patterns, leading to reduced consumption of nutrient‐dense foods such as fruits, vegetables, meat, and fish [24, 25]. Low‐income households, particularly those residing in urban informal settlements where daily wages constitute the primary source of income, have been disproportionately affected by food insecurity. Supply chain disruptions, market closures, and rising food costs have limited both the availability and affordability of diverse, nutritious foods, forcing households to rely on inexpensive staple foods with low nutrient content [26, 27, 28]. The Ukraine crisis has further intensified these challenges by driving up global food prices, forcing low‐income consumers to adopt less diverse and less nutritious diets [3, 17]. These dietary inadequacies have had significant implications for nutritional status, particularly among vulnerable populations. A study found that 54% of women living in Dhaka's slums were underweight, with a BMI below 18.5 [29], while another study among women aged 19 to 45 years in these settlements reported that 14% were underweight and only 5% were classified as overweight [30].

Despite the severity of these issues, research on food security, dietary diversity, and nutritional status among Dhaka's low‐income populations remains limited. Although some studies have examined aspects of food security and diet quality in urban low‐income communities [23, 24, 31, 32, 33], none have comprehensively addressed all three indicators in the context of the post‐COVID‐19 pandemic and the Russia–Ukraine crisis, with a particular focus on women in urban slums. To bridge this gap, this study aimed to assess the current status of food insecurity and diet quality as well as nutritional status among low‐income women in Dhaka City.

## 2 Materials and Methods

### 2.1 Study Design and Settings

A community‐based cross‐sectional study was conducted among residents of urban slums located within the Dhaka North City Corporation (DNCC) and Dhaka South City Corporation (DSCC). According to the Slum Census conducted by the Bangladesh Bureau of Statistics (BBS), a total of 13,938 slums were identified across all city corporations and municipalities in Bangladesh [34]. Among these, DNCC contained approximately 1644 slums housing around 469,696 people, while DSCC comprised about 1755 slums with an estimated 147,066 residents [34]. For this study, ten slums were conveniently selected from these two city corporations based on accessibility, safety considerations, and the availability of reliable population data. The selected sites included Bosila, Mohammadpur Bash Bari, and Korail from DNCC, and Demra, Komlapur Railway Track, Hazaribagh, Zigatola, Maniknagar, Shahjahanpur Malibagh, and Manda from DSCC.

### 2.2 Sampling Technique and Sample Size

We assumed a 50% prevalence of food insecurity to calculate the required sample size, as this conservative estimate yields the maximum sample size and ensures adequate statistical power for all three outcome variables: food insecurity, diet quality, and nutritional status. The sample size was determined using the widely applied formula: *n* = *Z* ^2^ × *p* (1 − p)/e^2^. Here, *n* represents the required sample size, *Z* (1.96) corresponds to the statistical value for a 95% confidence level, *p* (50%) indicates the estimated proportion, and e denotes the margin of error, set at 5% (0.05). The calculated sample size was 384, but to enhance representativeness, a total of 400 participants were included in the study. Participants were selected through convenience sampling.

### 2.3 Study Population

The study population consisted of low‐income women residing in selected slum areas of Dhaka City.

#### 2.3.1 Inclusion Criteria

Women residing in selected slum areas who had at least one child aged 6–59 months and provided informed consent to participate were included in the study.

#### 2.3.2 Exclusion Criteria

Women who were severely ill at the time of data collection or who declined to participate were excluded from the study.

### 2.4 Data Collection

Data were collected through home visits using a structured paper‐based questionnaire covering sociodemographic characteristics, food insecurity, and dietary intake. The questionnaire was translated into Bangla and back‐translated to ensure accuracy, and all interviews were conducted face‐to‐face in Bangla. Trained enumerators administered the survey following intensive instruction on questionnaire procedures, ethical considerations, and anthropometric measurement techniques. Supervisors reviewed completed questionnaires daily, and data were then cross‐checked and cleaned before analysis. Anthropometric measurements were obtained using a digital weight scale (TANITA HA 650, accurate to 0.1 kg) and a height scale (accurate to 0.1 cm); the weight scale was routinely calibrated. Data collection was conducted from September 1 to October 10, 2023.

### 2.5 Assessment of Food Insecurity and Coping Strategies

In this study, the Food Insecurity Experience Scale (FIES) was used to measure food insecurity at the individual level, reflecting each participant's personal experiences. FIES provides an experience‐based metric for measuring the severity of food insecurity (FI) among individuals or households [35]. This scale consists of eight questions that capture varying levels of food insecurity, with the first question addressing mild food insecurity and the final question reflecting severe food insecurity. Participants answered these questions using binary (yes or no) responses, which were aggregated into raw scores ranging from 0 to 8. Based on these scores, individuals were classified into three categories: food secure (FS) with a score of 0, moderate food insecurity (MFI) with scores from 1 to 6, and severe food insecurity (SFI) with scores from 7 to 8. Data from the FIES questionnaire were processed and analyzed using Microsoft Excel and an online platform called Shiny Apps.

Coping strategies were assessed through direct interviews using six structured questions focused on short‐term, food‐related responses to food insecurity. These strategies ranged from less severe actions, such as consuming cheaper or less preferred foods, to more severe ones, like reducing meal portions or skipping meals entirely. Respondents reported which strategies they had adopted, allowing for an understanding of the frequency and intensity of coping behaviors, following the framework proposed by Maxwell [36].

### 2.6 Assessment of Diet Quality

Diet quality among study participants was assessed using the Diet Quality Questionnaire (DQQ), a validated tool designed to evaluate dietary diversity and food group consumption patterns [37]. DQQ collects data on the consumption of specific food groups over the preceding 24 h, enabling the calculation of key indicators such as the Minimum Dietary Diversity for Women (MDD‐W), the Dietary Diversity Score (DDS), and the Global Dietary Recommendations (GDR) score, among others [38]. Dietary diversity among women was assessed using the MDD‐W indicator, as recommended by the Food and Agriculture Organization [39]. Participants who reported consuming at least five out of ten predefined food groups over the previous 24 h were considered to have adequate dietary diversity, indicating sufficient micronutrient intake. MDD‐W, all five food group indicators, the prevalence of consumption of food items associated with NCDs protection and NCDs risk, were calculated following the guidelines outlined in the DQQ indicator manual [38].

### 2.7 Assessment of Nutritional Status

Body mass index (BMI) was calculated by dividing the weight in kilograms by the square of the height in meters (kg/m^2^). Nutritional status categories were determined based on Asian‐specific BMI cut‐off [40]: underweight (BMI < 18.5 kg/m^2^), normal weight (BMI 18.5–22.9 kg/m^2^), overweight (BMI 23 to < 27.5 kg/m^2^), and obese (BMI ≥ 27.5 kg/m^2^).

### 2.8 Outcome Variables

In this study, food insecurity (moderate or severe insecurity, severe insecurity), dietary diversity based on MDD‐W (≥ 5 food groups, < 5 food groups), and nutritional status categorized by BMI (underweight, normal, overweight, and obese) were considered as primary outcome variables.

### 2.9 Independent Variables

In this study, we selected several predictor variables based on their established association with our outcome variables (food insecurity and MDDW) as reported in previous research [41, 42, 43, 44, 45, 46]. The predictor variables included slum location (Dhaka North City Corporation, Dhaka South City Corporation), age in years (≤ 24, 25–34, ≥ 35), employment status (unemployed, employed), education level (no formal education, primary incomplete: class 1–4, primary completed or higher: class 6 to above), household size (≤ 4 members, > 4 members), number of earning members (single, double or multiple), household monthly income (BDT ≤ 10,000, BDT 10,001–15,000, BDT > 15,000), and household monthly food expenditure (BDT ≤ 5000, BDT 5001–8,000, BDT > 8000).

### 2.10 Statistical Analysis

Descriptive analyses were performed to summarize food insecurity, diet quality, nutritional status, and background characteristics using frequencies and percentages. Chi‐square tests were conducted to examine associations between food insecurity and background characteristics, as well as between dietary diversity and background characteristics.

### 2.11 Regression Model Building

Two binary logistic regression models were constructed to examine key associations in the study. For the first model, food insecurity was treated as a binary outcome based on the FIES raw scores and categorized as ‘food secure’ (FIES score 0) and ‘food insecure’ (FIES score > 0) for regression analysis. This binary classification differed from the descriptive prevalence estimates reported for moderate and severe food insecurity. The model assessed the association between food insecurity and various sociodemographic characteristics. The second model evaluated the association between dietary diversity, measured by the MDD‐W (categorized as ≥ 5 food groups vs. < 5 food groups), and sociodemographic characteristics. Variables with *p*‐values < 0.25 in the bivariate analyses were considered for inclusion in the multivariable regression models [47]. Before finalizing the models, the assumptions of logistic regression were checked, and multicollinearity among independent variables was assessed using the Variance Inflation Factor (VIF), with values < 2 indicating no significant collinearity [48]. Associations were considered statistically significant at *p* < 0.05 and are presented as adjusted odds ratios (AOR) with 95% confidence intervals (CI). Additionally, the unadjusted associations (Crude Odds Ratios) are presented in Supplementary Tables 1 and 2. All analyses were performed using Statistical Package for Social Sciences (SPSS), version 26.0.

### 2.12 Ethical Considerations

The nature and objective of the study were clearly explained to all participants, and informed consent was obtained before their participation. Participation was voluntary, and confidentiality was ensured by removing all personal identifiers before analysis, using unique codes. Data were stored securely and accessible only to the research team. Ethical approval for the study was granted by the Institutional Review Board of the Faculty of Biological Sciences, University of Dhaka (Ref. No. 241/Biol.Scs).

## 3 Results

### 3.1 Basic Characteristics of the Participants

The sociodemographic characteristics and nutritional status of the study participants are presented in Table 1. The majority of the participants resided in the DSCC (67.5%; 270/400), and 70.3% (281/400) were aged 25–34 years. About 70% (279/400) of participants were employed, and 41% (163/400) had no formal education. The majority of households consisted of four or fewer members (61.8%). In terms of economic status, 39.8% (159/400) of households reported a monthly income of BDT ≤ 10,000, while the most common range for monthly food expenditure was BDT 5001–8000 (42%). Regarding nutritional status, 42.3% (169/400) of the participants were classified as overweight or obese, and 8.8% (35/400) were underweight.

**Table 1:** Sociodemographic characteristics and nutritional status of the study participants (*N* = 400).

Characteristics	Frequency	Percent
Location of the Slum		
*North City Corporation*	130	32.5
South City Corporation	270	67.5
Age (years)		
≤ 24	60	15.0
25–34	281	70.3
≥ 35	59	14.8
Employment status		
Unemployed	121	30.3
Employed	279	69.8
Educational level		
No formal education	163	40.8
Primary incomplete	157	39.3
Primary completed or higher	80	20.0
Household size		
≤ 4	247	61.8
> 4	153	38.3
Household Earning member		
Single	187	46.8
Double or multiple	213	53.3
Household monthly income (BDT)		
≤ 10000	159	39.8
10001–15000	120	30.0
> 15000	121	30.3
Monthly food expenditure (BDT)		
≤ 5000	139	34.8
5001–8000	168	42.0
> 8000	93	23.3
Nutritional status		
Underweight (BMI < 18.5)	35	8.8
Normal (BMI 18.5–22.9)	196	49.0
Overweight (BMI 23 to < 27.5)	136	34.0
Obese (BMI ≥ 27.5)	33	8.3

*Note:* BMI was classified based on Asian‐specific BMI cut‐off.

Abbreviations: BDT, Bangladeshi Taka, 1 USD = 110.22 BDT, BMI = Body Mass Index.

### 3.2 Food Insecurity Prevalence Among the Participants

**Figure 1:**
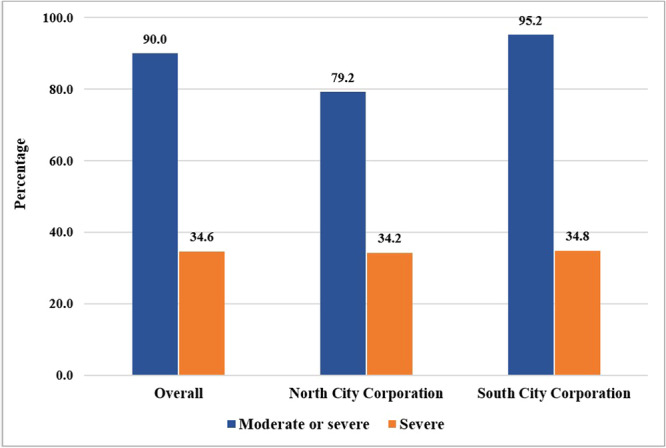
Food insecurity among the study participants.

Overall, 90% (360/400) of participants experienced moderate or severe food insecurity, with 34.6% (139/400) classified as severely food insecure. In the DNCC, 79.2% (103/130) reported moderate or severe food insecurity, while 34.2% (44/130) experienced severe food insecurity. In contrast, food insecurity was more prevalent in the DSCC, where 95.2% (257/270) of participants experienced moderate or severe food insecurity, and 34.8% (95/270) faced severe food insecurity (Figure 1).

### 3.3 Adopted Coping Strategies by the Participants

**Figure 2:**
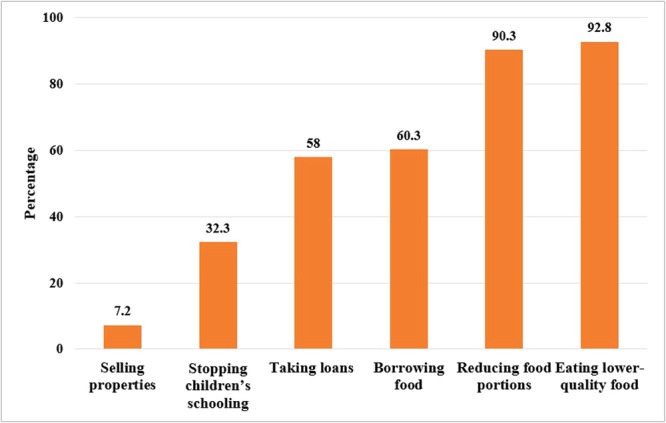
Coping strategies adopted by study participants.

Figure 2 shows the coping strategies adopted by study participants in response to food insecurity. The most common strategies were eating lower‐quality food (92.8%; 371/400) and reducing food portions (90.3%; 361/400), followed by borrowing food (60.3%; 241/400) and taking loans (58%; 232/400). Less frequently reported strategies included stopping children's schooling (32.3%; 129/400) and selling properties (7.2%; 29/400).

### 3.4 Dietary Quality Among the Participants

**Figure 3:**
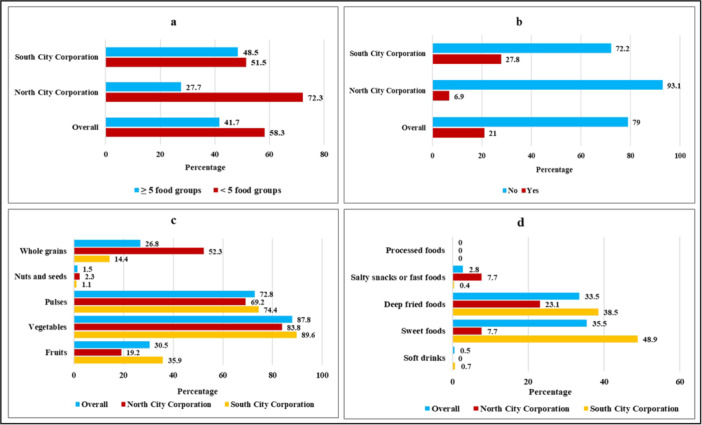
Diet quality indicators (a) MDD‐W, (b) Consumption of all five recommended food groups, (c) Consumption of NCD protective, and (d) Consumption of NCD risk food groups) among the study participants.

Figure 3 illustrates diet quality indicators among study participants, stratified by location. Overall, 58.3% (233/400) of participants consumed fewer than five food groups, indicating inadequate dietary diversity. Dietary diversity was significantly lower among participants from DNCC compared to DSCC (27.7% vs. 48.5%, *p* < 0.001) (Figure 3a). Only 21% (84/400) of participants consumed all five recommended food groups, with a significantly lower proportion in DNCC than in DSCC (6.9% vs. 27.8%, *p* < 0.001) (Figure 3b). Among NCD‐protective foods, vegetable consumption was the highest (87.8%), followed by pulses (72.8%) and fruits (30.5%). In contrast, the intake of nuts and seeds (1.5%) and whole grains (26.8%) was notably low (Figure 3c). Regarding NCD‐risk foods, sweet foods (35.5%) and deep‐fried foods (33.5%) were the most commonly consumed, while processed foods were not consumed (Figure 3d).

### 3.5 Factors Associated With Food Insecurity

Factors associated with food insecurity among participants are presented in Table 2. The frequencies presented in this table represent the binary food insecurity classification (food secure vs. food insecure) derived from the FIES raw scores and used for the logistic regression analysis, which differs from the prevalence estimates of moderate and severe food insecurity reported in Table 1. Slum location was a major determinant, with individuals residing in DSCC having significantly higher odds of food insecurity compared to those in DNCC (AOR = 23.2, 95% CI: 4.5–117.6, *p* < 0.001). The number of household earning members was also significant, as households with multiple earners were less likely to experience food insecurity (AOR = 0.19, 95% CI: 0.06–0.5, *p* = 0.038) than those with a single earner. However, age, education level, employment status, and household income were not significantly associated with food insecurity. Additionally, the unadjusted associations (Crude Odds Ratios) are presented in Supplementary Table 1.

**Table 2:** Factors associated with food insecurity among study participants (*N* = 400).

Characteristics	Food insecurity *n* (%)	AOR (95% CI)	*p*‐value
Location of the slum			
North City Corporation	111 (29.3)	1	
South City Corporation	268 (70.7)	23.2 (4.5, 117.6)	*p* < 0.001
Age in years			
≤ 24	58 (15.1)	1	
25–34	265 (69.9)	0.36 (0.09, 1.4)	*p* = 0.15
≥ 35	57 (15)	0.96 (0.1, 6.8)	*p* = 0.97
Education			
No formal education	151 (39.8)	1	
Primary incomplete	155 (40.9)	3.3 (0.7, 16.3)	*p* = 0.14
Primary completed or higher	73 (19.3)	0.3 (0.09, 1.1)	*p* = 0.07
Employment status			
Unemployed	107 (28.2)	1	
Employed	272 (71.8)	1.5 (0.4, 5.08)	*p* = 0.49
Household earning member			
Single	209 (55.1)	1	
Double or multiple	170 (44.9)	0.19 (0.06, 0.5)	*p* = 0.03
Household monthly income (BDT)			
≤ 10,000	148 (39.1)	1	
10,001–15,000	114 (30.0)	1.09 (0.34, 3.5)	*p* = 0.88
> 15,000	117 (30.9)	0.9 (0.2, 4.1)	*p* = 0.98

Abbreviations: AOR, Adjusted odds ratio; BDT, Bangladeshi Taka, 1 USD = 110.22 BDT; CI, Confidence interval.

### 3.6 Factors Associated With Dietary Diversity

Table 3 presents the factors associated with achieving adequate dietary diversity (MDD‐W) among study participants. Individuals aged 25–34 years had significantly higher odds of meeting the minimum dietary diversity compared to those aged ≤ 24 years (AOR = 2.5, 95% CI: 1.1–5.3, *p* = 0.016). Education level was a key determinant; participants with incomplete primary education (AOR = 3.8, 95% CI: 2.1–6.6, *p* < 0.001) and those with completed primary or higher education (AOR = 3.2, 95% CI: 1.6–6.3, *p* < 0.001) were significantly more likely to achieve adequate dietary diversity than those with no formal education. Households with multiple earning members had greater odds of adequate dietary diversity (AOR = 1.8, 95% CI: 1.2–2.8, *p* < 0.001) compared to single‐earner households. Similarly, a monthly household income exceeding BDT 15,000 was strongly associated with adequate dietary diversity (AOR = 5.5, *p* < 0.001) relative to households earning BDT 10,000 or less. Additionally, households spending between BDT 5001 and 8000 on food were more likely to meet the dietary diversity threshold (AOR = 2.0, 95% CI: 1.06–3.7, *p* = 0.030). The unadjusted estimates (Crude Odds Ratios) are presented in Supplementary Table 2.

**Table 3:** Factors associated with dietary diversity (MDD‐W) among study participants (*N* = 400).

Characteristics	Adequate dietary diversity (≥ 5 food groups) *n* (%)	AOR (95% CI)	*p*‐value
Location of the slum			
North City Corporation	36 (21.6)	1	
South City Corporation	131 (78.4)	1.4 (0.8, 2.6)	*p* = 0.21
Age in years			
≤ 24	13 (7.8)	1	
25–34	132 (79.0)	2.5 (1.1, 5.3)	*p* = 0.02
≥ 35	22 (13.2)	1.9 (0.7, 4.9)	*p* = 0.16
Education status			
No education	32 (19.2)	1	
Primary incomplete	93 (55.7)	3.8 (2.1, 6.6)	*p* < 0.001
Primary completed or higher	42 (25.1)	3.2 (1.6, 6.3)	*p* < 0.001
Employment status			
Unemployed	35 (21.0)	1	
Employed	132 (79.0)	1.4 (0.7, 3)	*p* = 0.27
Household earning member			
Single	63 (37.7)	1	
Double or multiple	104 (62.3)	1.8 (1.2, 2.8)	*p* < 0.001
Household monthly income (BDT)			
≤ 10,000	35 (21.0)	1	
10,001–15,000	48 (28.7)	1.6 (0.8, 3.2)	*p* = 0.10
> 15,000	84 (50.3)	5.5 (2.5, 12)	*p* < 0.001
Household food expenditure (BDT)			
≤ 5000	30 (18.0)	1	
5001–8000	82 (49.1)	2.0 (1.06, 3.7)	*p* = 0.03
> 8000	55 (32.9)	1.6 (0.7, 3.8)	*p* = 0.24

Abbreviations: AOR, Adjusted odds ratio; BDT, Bangladeshi Taka, 1 USD = 110.22 BDT; CI, Confidence interval.

## 4 Discussion

This study explored the food security status, dietary quality, and nutritional status of women residing in low‐income slum communities in Dhaka City, Bangladesh. The study revealed a high prevalence of food insecurity, with the vast majority of participants experiencing some level of it; among them, over one‐third were classified as severely food insecure. Participants residing in the DSCC were found to have significantly higher odds of experiencing food insecurity. In contrast, households with a greater number of earning members demonstrated significantly lower odds of food insecurity. More than half of the women failed to meet the MDD‐W criteria. Women aged 25–34 years, those with higher educational attainment, and those from households with multiple earners, higher income, and greater food expenditures were significantly more likely to achieve adequate dietary diversity. Among foods linked to the prevention of non‐communicable diseases (NCDs), vegetables and pulses were the most commonly consumed items. Regarding NCD‐risk foods, sweet foods and deep‐fried items were consumed most frequently. A dual burden of malnutrition was observed, with 8.8% of women classified as underweight and 42.3% as overweight or obese.

Our study found that nine in ten low‐income women experienced moderate or severe food insecurity, and one in three faced severe food insecurity. These findings are consistent with a previous study indicating that the majority of low‐income households (93.2%) in urban slum‐like areas of Bangladesh faced food insecurity [49]. Similarly, a survey by the World Food Program (WFP) highlighted the severity of food insecurity in Dhaka's urban slums, where vulnerable populations struggle with limited income and high living costs [50]. However, our reported prevalence is substantially higher than the national estimate of 24% provided by the Integrated Food Insecurity Phase Classification [16]. Furthermore, our findings indicate a considerably higher prevalence compared to similar urban settings in neighboring countries such as India (36.1%) [51] and Nepal (46%) [52], where food insecurity remains a significant concern. This disparity suggests that food insecurity disproportionately affects certain urban populations, particularly those in low‐income settlements.

Regional disparities in food insecurity were also observed in our study, as participants from the DSCC had significantly higher odds of experiencing food insecurity compared to those in DNCC. This difference may be attributed to higher population density, poorer housing conditions, and limited access to essential services in DSCC, particularly in slum areas. These factors likely exacerbate vulnerability to food insecurity. In contrast, DNCC benefits from relatively better infrastructure and living conditions, which may offer greater resilience against food insecurity. Consistent with our findings, a recent study also reported a higher prevalence of food insecurity in Dhaka South [53]. The higher population density in DSCC compared to DNCC may intensify competition for limited food resources. Additionally, global food supply disruptions have reduced food availability nationwide, leading to higher prices. Consequently, increased demand amid limited supply has pushed food prices beyond the reach of many low‐income households, particularly in DSCC.

Households with two or more income earners were significantly less likely to experience food insecurity than single‐earner households. This finding aligns with existing research, which emphasizes that multiple income sources enhance financial stability and reduce vulnerability to food insecurity [54, 55].

Beyond food security, dietary diversity was also a major concern, as our study found that 58.3% of participants did not meet the MDDW criteria. This highlights a serious risk of micronutrient deficiencies among women in urban slum settings. Our findings are in line with a previous study reporting that 59.9% of women from a low‐income, slum‐like urban area in Bangladesh failed to meet the MDDW threshold [49]. Similar trends have been observed in other low‐income settings, such as a study in Pune, India, where only 36.5% of pregnant slum‐dwelling women met the dietary diversity criteria [56].

In our study, age played a key role in dietary diversity, as women aged 25–34 years demonstrated higher dietary diversity compared to younger age groups. This trend may be due to greater autonomy in food choices, increased nutritional awareness, and improved financial stability as individuals grow older.

Consistent with previous studies, this study found a positive association between higher education levels and greater dietary diversity [44, 57]. This association can be attributed to the role of education in improving nutritional knowledge, raising health awareness, and empowering individuals to make informed food choices—factors that collectively promote healthier eating habits and greater dietary diversity.

Our study found that households with multiple earners and higher incomes were more likely to achieve adequate dietary diversity compared to single‐earner and lower‐income households. These findings are consistent with previous studies showing that women from wealthier households tend to have better dietary diversity [44, 57]. This is also supported by economic theory, which suggests that higher income improves food access and choice, allowing households to afford nutrient‐dense foods such as fruits, vegetables, and animal‐source products [58]. Moreover, multiple income sources enhance financial stability, reducing food insecurity and fostering better dietary diversity [59].

Food expenditure was also a key determinant of dietary diversity. This study found that households with higher food expenditures had significantly greater dietary diversity compared to those with lower food expenditures. This finding aligns with a study in rural Bangladesh, which also reported a strong link between food expenditure and dietary diversity [60]. Increased food expenditures likely allow households to access a wider range of food groups, including protein‐rich and micronutrient‐dense options, thereby improving dietary quality [61].

Regarding food consumption patterns, vegetables and pulses were the most frequently consumed NCD‐protective foods among participants, while fruit intake was moderate, and whole grains and nuts/seeds were the least consumed. This pattern may be attributed to limited access to healthier food options, as individuals in low‐resource settings often prioritize affordable and locally available nutritious foods such as vegetables and pulses. The moderate consumption of fruits suggests some level of awareness regarding their health benefits, but it may also reflect financial constraints and seasonal variability in availability. In contrast, the low intake of whole grains and nuts/seeds—key components for reducing NCD risks—highlights significant barriers, including cost, limited availability, and insufficient awareness of their benefits. Regarding NCD‐risk foods, sweet foods and deep‐fried items were the most frequently consumed by the participants. This trend may be driven by the preference for energy‐dense, low‐cost foods in economically constrained communities, which can contribute to increased NCD risks.

The present study also highlights the dual burden of malnutrition, with 8.8% of women classified as underweight and 42.3% as overweight or obese. These findings reflect global trends, where overnutrition is rising alongside persistent undernutrition [62]. A national survey‐based study similarly reported a high prevalence (55.5%) of overweight and obesity among women of reproductive age in Bangladesh, supporting the growing concern of overnutrition in the country [63]. Another study among women aged 19–45 years living in Dhaka's slums reported that 14% were underweight, while only 5% were overweight [30]. While the prevalence of undernutrition in our study closely aligns with this previous study, the prevalence of overnutrition is notably higher. This discrepancy may be attributed to the increasing trend of overweight and obesity in urban settings, driven by dietary transitions, sedentary lifestyles, and greater access to energy‐dense foods.

Alongside the nutritional challenges identified, urban poverty and vulnerability in Bangladesh have deepened amid rapid urbanization and rural‐to‐urban migration [64]. Cities like Dhaka have become both economic hubs and pockets of concentrated poverty, where rising living costs and precarious livelihoods heighten food insecurity. Yet, social protection coverage has not kept pace with these shifts. Bangladesh's safety net system remains largely rural‐focused—of over 100 programs in 2024–25, only 23 specifically target urban populations, representing just 4.1% of the total social protection budget [64]. Consequently, coverage in urban areas (24%) remains far below that in rural areas (44%) [64], revealing persistent targeting gaps and limited inclusion of the urban poor.

### 4.1 Policy Implications

Policy measures should prioritize expanding targeted social safety nets and subsidized food programs in urban slums, particularly in the Dhaka South City Corporation, to improve access to nutrient‐rich foods among food‐insecure households. Income‐generating opportunities, especially for women, should be promoted to enhance household purchasing power. Community‐based nutrition education campaigns focusing on dietary diversity and the inclusion of fruits, whole grains, and nuts are essential, alongside regulatory actions to limit the availability and marketing of ultra‐processed, NCD‐risk foods. Multi‐sectoral collaboration between health, food, urban planning, and social welfare sectors is critical to integrate nutrition‐sensitive interventions into urban development strategies. Regular nutrition surveillance and research in low‐income communities should be supported to ensure evidence‐based planning and adaptive policy responses.

### 4.2 Strengths and Limitations

This study provides valuable insights into food security, dietary quality, and nutritional status among women residing in Dhaka's urban slums, a vulnerable population often underrepresented in research. The use of validated tools such as the FIES and DQQ enhances the reliability of data on food insecurity and diet quality. However, the cross‐sectional design limits the ability to establish causal relationships. Although data were collected from multiple slum areas across different parts of Dhaka City to minimize potential bias and ensure variability among participants, the use of convenience sampling may still introduce selection bias, which could affect the generalizability of the findings. Additionally, self‐reported dietary data may be subject to recall and social desirability biases. Despite these limitations, this study provides critical evidence to inform policies and interventions aimed at improving the well‐being of vulnerable populations in Bangladesh.

## 5 Conclusion

This study shows critical public health challenges, including the alarming prevalence of food insecurity, poor diet quality, and the coexistence of undernutrition and overweight/obesity among women living in low‐income settlements of Dhaka City. Nearly one‐third of participants experienced severe food insecurity, about three in five women had inadequately diversified diets, and almost half were affected by malnutrition. Addressing these issues requires expanding social safety nets, subsidized food programs, and income‐generating opportunities for vulnerable households, alongside targeted nutrition education promoting dietary diversity and reduced intake of NCD‐risk foods. Multi‐sectoral collaboration and continued research are essential for developing sustainable, context‐specific strategies to improve food security and nutritional well‐being in these urban settings.

## Author Contributions

**Afsana Rahaman Munmun:** conceptualization, data curation, investigation, writing – original draft. **Md Shahadoth Hossain:** conceptualization, methodology, software, data curation, investigation, formal analysis, writing – review and editing. **Sneha Sarwar:** conceptualization, investigation, writing – original draft, writing – review and editing, methodology. **Md Mahbub Alam:** conceptualization, investigation, formal analysis, methodology, writing – review and editing, data curation. **Md Jarif Mahbub:** writing – original draft, writing – review and editing, conceptualization, methodology, investigation. **Most Nishat Anam:** conceptualization, methodology, writing – original draft, investigation, data curation. **Zinnia Tasnim:** conceptualization, writing – original draft, investigation, writing – review and editing. **Farah Ahmed:** conceptualization, investigation, writing – original draft, writing – review and editing. **Md Ruhul Amin:** conceptualization, methodology, supervision, investigation, writing – review and editing, data curation.

## Funding

The authors have nothing to report.

## Ethics Statement

Ethical approval for the study was obtained from the Institutional Review Board of the Faculty of Biological Sciences, University of Dhaka (Ref. No. 241/Biol. Scs).

## Consent

The nature and objectives of the study were clearly explained to all participants. Participation was entirely voluntary, and informed consent was obtained prior to their involvement in the study.

## Conflicts of Interest

The authors declare no conflicts of interest.

## Transparency Statement

The lead author, Md. Ruhul Amin affirms that this manuscript is an honest, accurate, and transparent account of the study being reported; that no important aspects of the study have been omitted; and that any discrepancies from the study as planned (and, if relevant, registered) have been explained.

## Supporting information


**Table S1:** Factors associated with food insecurity among study participants (Unadjusted estimates).
**Table S2:** Factors associated with dietary diversity (MDDW) among study participants (Unadjusted estimates).

## Data Availability

The data that support the findings of this study are available on request from the corresponding author. The data are not publicly available due to privacy or ethical restrictions.
