# Supplementary material for: Food Insecurity and Diet Quality Among Low‐Income Women in Dhaka City Amidst COVID‐19 and the Ukraine Crisis: A Cross‐Sectional Study
Source: Health Sci Rep. 2026 Aug 4;9(8):e72980. doi: 10.1002/hsr2.72980 (PMC13438890; doi:10.1002/hsr2.72980)
Supplement: Supplementary file 1 [file HSR2-9-e72980-s001.docx]

**Supplementary tables:**

**S. Table 1.** Factors associated with food insecurity among study participants (Unadjusted estimates)

| **Characteristics** | **COR (95% CI)** | ***p*-value** |
| --- | --- | --- |
| ***Location of the Slum*** |  |  |
| North City Corporation | 1 |  |
| South City Corporation | 22.9 (5.2, 100) | < 0.001 |
| ***Age in years*** |  |  |
| 15-24 | 1 |  |
| 25-34 | 0.8 (0.2, 3.09) | 0.832 |
| ≥ 35 | 1.5 (0.2, 9.3) | 0.663 |
| ***Education*** |  |  |
| No formal education | 1 |  |
| Primary incomplete | 6.1 (1.3, 27.9) | 0.019 |
| Primary completed or higher | 0.8 (0.3, 2.1) | 0.705 |
| ***Employment status*** |  |  |
| Unemployed | 1 |  |
| Employed | 5.0 (1.9, 12.9) | 0.001 |
| ***Household earning member*** |  |  |
| Single | 1 |  |
| Double or multiple | 0.36 (0.16, 0.81) | 0.014 |
| ***Household monthly income*** |  |  |
| ≤ 10000 | 1 |  |
| 10001-15000 | 1.4 (0.5, 3.9) | 0.509 |
| > 15000 | 2.1 (0.6, 7.0) | 0.193 |

***Note:*** COR: Crude odds ratio, CI: Confidence interval, Food insecurity was treated as a binary outcome based on the FIES raw scores and categorized as ‘food secure and food insecure’ for regression analysis.

**S. Table 2.** Factors associated with dietary diversity (MDDW) among study participants (Unadjusted estimates)

| **Characteristics** | **COR (95% CI)** | ***p*-value** |
| --- | --- | --- |
| ***Location of the slum*** |  |  |
| North City Corporation | 1 |  |
| South City Corporation | 2.4 (1.5, 3.8) | < 0.001 |
| ***Age in years*** |  |  |
| 15 to 24 | 1 |  |
| 25 to 34 | 3.2 (1.6, 6.1) | 0.001 |
| ≥ 35 | 2.1 (0.9, 4.8) | 0.064 |
| ***Education status*** |  |  |
| No education | 1 |  |
| Primary incomplete | 5.9 (3.6, 9.8) | < 0.001 |
| Primary completed or higher | 4.5 (2.5, 8.1) | < 0.001 |
| ***Employment status*** |  |  |
| Unemployed | 1 |  |
| Employed | 2.2 (1.3, 3.4) | 0.001 |
| ***Household earning member*** |  |  |
| Single | 1 |  |
| Double or multiple | 1.8 (1.2, 2.8) | 0.002 |
| ***Household monthly income*** |  |  |
| ≤ 10000 | 1 |  |
| 10001-15000 | 2.3 (1.3, 3.9) | 0.001 |
| > 15000 | 8.0 (4.6, 13.7) | < 0.001 |
| ***Household food expenditure*** |  |  |
| ≤ 5000 | 1 |  |
| 5001-8000 | 3.4 (2.0, 5.7) | < 0.001 |
| > 8000 | 5.2 (2.9, 9.3) | < 0.001 |

***Note:*** COR: Crude odds ratio, CI: Confidence interval, Dietary diversity was measured by the MDD-W (categorized as ≥ 5 food groups vs. < 5 food groups).
